# An ELISA-based procedure for assaying proteins in digests of human leukocytes and cell lines, using specifically selected peptides and appropriate antibodies

**DOI:** 10.1186/1477-5956-4-14

**Published:** 2006-06-21

**Authors:** Ori Braitbard, Janette Bishara-Shieban, Hava Glickstein, Miriam Kott-Gutkowski, Umberto Pace, Deborah G Rund, Wilfred D Stein

**Affiliations:** 1 Biological Chemistry, Silberman Institute of Life Sciences, Hebrew University, Jerusalem, Israel; 2 MDR Tests Ltd, 28 Pierre Koenig St, Jerusalem, Israel; 3 Hematology Department of Hadassah University Hospital, Jerusalem, Israel

## Abstract

**Background:**

We describe the application of an ELISA-based assay (the Peptidomatrix) that can be used to simultaneously identify and quantitate a number of proteins in biological samples. The biological sample (blood component, biopsy, culture or other) is first lysed to release all the proteins, without any additional separation. The denatured proteins in the sample are then digested in bulk with the desired proteolytic enzyme(s). The peptides in the digest are then assayed by appropriate antibodies, using a competition ELISA protocol.

**Results:**

As an example of its use, the present paper applies the Peptidomatrix to the assay of four membrane proteins MDR1 (P-glycoprotein or ABCB1), MRP1 (ABCC1), BCRP/MXR (ABCG2) and the alpha subunit of the Na, K_ATPase (ATP1A1), present in a number of cell lines and in human lymphocytes. We show that we can detect and quantitate these proteins, using a series of peptide-antibody pairs, and that we can differentiate between cell lines or cell preparations that express the target proteins and those that do not.

**Conclusion:**

We have devised a simple, ELISA-based proteomics assay that enables the quantitation of designated proteins in a cell or tissue sample, and that can be used in any laboratory, with minimal specialized equipment.

## Background

The revolution in biology initiated by the Genome Project is being further stimulated by research aimed at the elucidation of the proteome, the complement of proteins expressed by an organism [1]. Proteomics aims to develop methods for providing a total accounting of the proteins present in a biological sample, with all the valuable insights that will flow from achieving this aim [2-7] Much proteomics research employs techniques such as 2D gel electrophoresis for the separation of the protein mixtures followed by the use of HPLC and mass spectroscopy technology for the identification of the target proteins [7,4]. However, there is a definite need for simpler and less costly methods that can identify a limited number of proteins in a biological sample, as needed in small clinical or research laboratories.

In this paper we describe an ELISA-based assay, the Peptidomatrix, based on a procedure which has been developed to identify and quantitate proteins in biopsies and other biological samples[8,9]. Since the principle of the assay is the use of peptides derived from a tryptic digest of the sample, it can be used on samples that have undergone denaturation. Thus, an advantage of the Peptidomatrix is that the procedure does not require that the target protein be present in its native form. In addition, no prior isolation and purification of the protein target is required for setting up the assay. All that is needed is knowledge of the sequence of the protein (or of the mRNA that codes for it).

The biological sample (blood fraction, biopsy, culture or other) is first lysed to release all the proteins, without any additional separation. The denatured proteins in the sample are then digested in bulk with the desired proteolytic enzyme(s). The peptides in the digest are then assayed by appropriate antibodies, using a competition ELISA protocol.

The Peptidomatrix assay is based on competition between a peptide derived from a proteolytic digest of the sample and an identical synthetic peptide, which has been pre-bound to the ELISA plate, for an appropriate antibody[10].

## Results

The Peptidomatrix assay uses peptides that are chosen as being (i) specific for a target protein and (ii) present amongst the products of tryptic digestion of that protein. We subjected the membrane protein transporters MDR1 (or ABCB1 i.e. P-glycoprotein 1), MXR (or BCRP i.e. ABCG2) and MRP1 (ABCC1) the alpha chain of Na, K-ATPase (ATP1A1) to a virtual tryptic digestion and from the products of digestion, we selected the peptides of length 7 to 15 amino-acids. Each one of these peptides was analyzed using the BLAST program (see methods). A desirable peptide contains only matches that are 5 amino acids or shorter, and a minimal number of them. The peptides chosen are listed in Table 1.

**Table 1:** The peptides and antibodies used throughout this study. Note that all these peptides have a cysteine at their N terminus, which has been added for conjugating them to a carrier protein for the immunization.

Peptide name	Sequences	Location	Serum from rabbit
MDR1 P1	**C**QDVSWFDDPK	799–808	#64840, #64841
MDR1 P3	**C**SEIDALEMSSNDSR	646–659	#65158, #64541
MDR1 P494	MPNTLEGNVT**K**	1027–1036	C494 (Commercial monoclonal antibody)
			
MXR P1	**C**VGTQFIR	178–184	#65158, #64541
MXR P2	**C**LAEIYVNSSFYK	332–343	#64795, #64850
MXR P3	**C**EISYTTSFCHQLR	366–378	#64795, #64850
MXR P4	**C**LFIHYISGYYR	454–465	#64853, #64851
MXR P5	**C**NDSTGIQNR	418–426	#64853, #64851
			
MRP1 P1	**C**PSDLLQQR	1511–1518	#A0151, #A0152
MRP1 P2	**C**DLWSLNK	240–246	#A0151, #A0152
			
NaK ATPase P1	**C**IPFNSTNK	478–485	#64845, #64846
NaK ATPase P2	**C**PTTPEWVK	74–81	#64845, #64846
NaK ATPase P3	**C**TGTLTQNR	368–375	#64855, #64856
NaK ATPase P4	**C**YEPAAVSE	11–07	#64855, #64856

**Figure 1:**
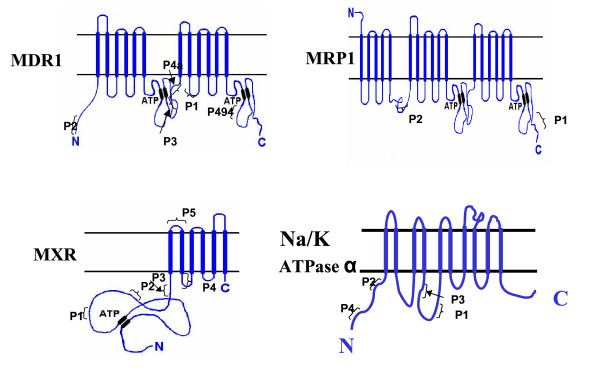
**Structure of the proteins detected by the Peptidomatrix**. The ATP binding site for the ABC transporters and the location of the peptides selected for the Peptidomatrix are indicated.

The positions of these peptides in the primary sequence of each of the four proteins are shown in Fig 1. Polyclonal antibodies were generated in rabbits for all of these peptides except in the case of the peptide denoted as P494 for which a commercial monoclonal antibody (C494) was available.

**Figure 2:**
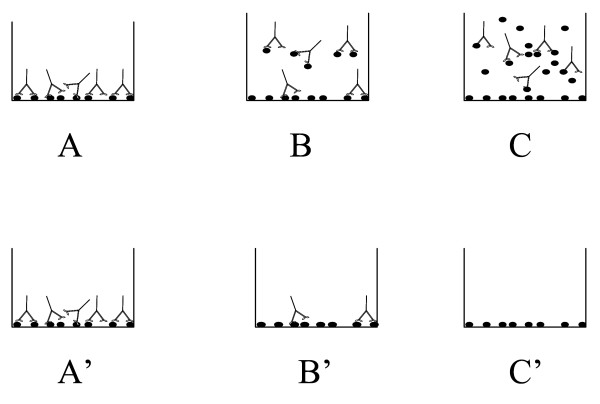
**The procedure of the 'Peptidomatrix' assay**. The ELISA Peptidomatrix is a competition assay: Peptides are bound to the plastic wells and they are subsequently exposed to antibodies generated against them. A labeled secondary antibody is used to detect the bound primary antibodies. In the assay itself the antibodies are mixed with soluble synthetic peptide in known amounts, to generate a calibration curve. The peptide in solution competes with the bound peptide and will affect the signal. As shown schematically in the figure: Panel A, A': No free peptide is added, thus the signal will be the highest. Panel B, B': A small amount of free peptide is added and the signal will be intermediate. Panel C, C': A large amount of competing peptide is added, thus no antibody binds to the well and the signal is minimal.

The Peptidomatrix assay, as described in the Introduction, is a competition assay (Figure 2): Peptides are bound to plastic wells. Antibodies specific to the peptide are then added in solution and allowed to bind to the attached peptide in the presence or the absence of a sample digest. A calibration curve is generated in parallel with known quantities of free synthetic peptide. The concentration of soluble peptide in the sample is then measured by interpolation.

**Figure 3:**
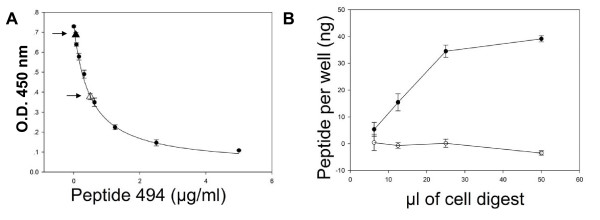
**Detection of the MDR1 peptide P494**. In panel A calibration curves are plotted as optical density (OD450) vs. peptide concentration. The curve is the calculated regression fit, based on the hyperbolic equation detailed in the Materials and Methods section. The OD values of 50 μl of cell digest (approximately 4.5 million cells) from wild type and drug-resistant CEM cells are shown as solid and empty triangles respectively. For the sake of clarity we have added arrows to indicate the position of these points on the curve. In panels B the amounts of peptide (in nanograms) for a range of digest volumes (from 6.25 μl to 50 μl) are shown. These values have been calculated using the regression curve of panels A.

Figure 3 shows a typical experiment in which peptide P494 (from MDR1) and monoclonal antibody C494 were used to detect and quantify the peptide in CEM cells, in digests prepared from either wild-type cells (WT) or from multidrug resistant cells (Col1000). In panel A the calibration curve depicting the ELISA signal as function of free synthetic peptide is shown as a solid line. This curve is a descending hyperbola, since the more free peptide is added the lower the signal. In parallel, aliquots of the cell digest were assayed in the same way. Also in this case, the more peptide present in the digest, the lower the signal will be. In Figure 3A the signals from digests of WT and Col1000 CEM cells are shown (solid and empty triangles). The concentration of peptide in the digest is calculated by interpolation on the calibration curve. A simple calculation (see Materials and Methods) yields an estimate of the concentration of peptide (in ng/ml) present in the digest. Panel B of Fig 3 summarizes the results of several experiments such as those shown in panel A. In each experiment, we added increasing amounts of the tryptic digest of the wild type or drug resistant CEM cells, estimated the amounts of peptide in the sample and plotted these against the amount of added digest. The amount of peptide detected in the drug resistant cells increases with increasing amounts of digest while the level in the wild type CEM cells remains close to zero.

**Figure 4:**
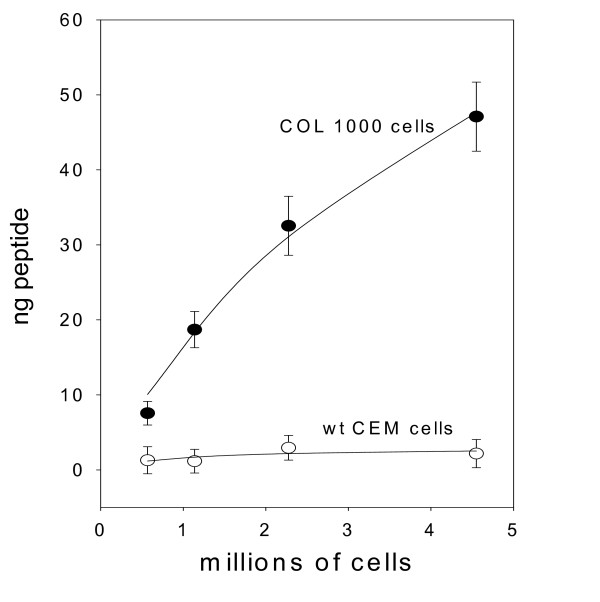
**Average (± SE) of 15 experiments detecting P494 in CEM cells**. The abscissa values have been recalculated in millions of cells, in order to deduce the number of PgP molecules per cell (see text for details).

Fig 4 depicts the means and standard errors of 15 experiments performed as in Fig 3, with the data now presented as ng of peptide detected per millions of cells. There is a very clear difference between the data set for the drug resistant Col1000 cells and the parent CEM cells. The plot for the Col1000 cells is not linear, presumably because the wells hold only a limited amount of the bound peptide. Fitting these data to a hyperbola (i.e. Michaelis-Menten equation) gave an estimate of the initial slope of 19.7 ± 2.5 ng peptide per million cells and a half-saturation value of 5.2 ± 1.7 million cells. With a molecular weight of 1158.31 for the peptide, this slope translates to 10.2 million P-glycoprotein molecules per Col1000 cell.

Interestingly, titration curves such as Fig 3A enable another estimate to be made of the number of P-glycoprotein molecules per Col1000 cell. The average of the half-saturation values for the 15 calibration curves performed to generate Fig 4 was 0.678 ± 0.0678 (SE, n = 15) μg/ml peptide. Since each well receives 100 μl of peptide solution, there is 68 ± 6.8 ng peptide in the average well at which the antibody is one-half saturated. In Fig 4, half-saturation is reached at the peptide present in 5.2 ± 1.7 million cells. Equating these two values, one can conclude that there are 68/5.2 or 13.0 ± 4.4 ng peptide per million cells (or 6.7 million P-glycoprotein molecules per Col1000 cell). This and the estimate of 19.7 ± 2.5 ng per well found from the initial slope of the plot of Fig 4 are less than 1 SE unit apart.

**Figure 5:**
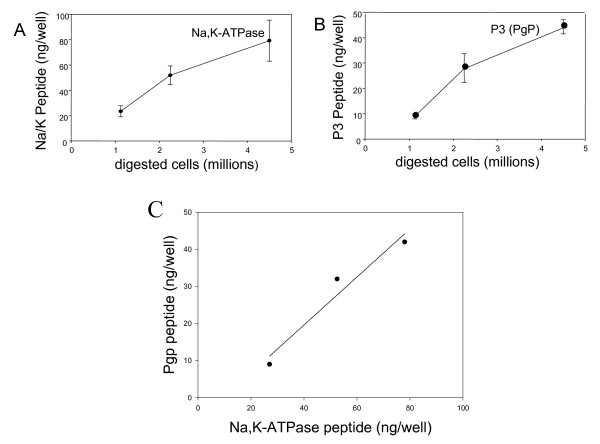
**Simultaneous detection of Na/K ATPase and MDR1 proteins in cells from a leukemia patient**. In Panel A and B the measured amounts of the peptides PgP P3 and ATPase P4 (in ng/well) are plotted as function of the amounts of cells. In panel C the same calculated amounts of peptides are plotted one against the other, allowing the calculation of the ATPase to PgP ratio over a range of cell amounts.

We chose to apply the Peptidomatrix protocol to the Na, K-ATPase in order to provide a "calibrating protein" that would be present in every animal cell type and would enable the Peptidomatrix user to normalize data in terms of this protein. It is also a fitting control in this case since this protein share a large similarity in structure and cellular location with the other target proteins. Fig 5 depicts such an experiment in which, on the same ELISA plate, samples of a digest of leukemia cells (from patient ME) were assayed for MDR1 using the P3 peptide and the appropriate polyclonal antibody and, in parallel, were assayed for the Na, K-ATPase using the P4 peptide and appropriate polyclonal antibody. Parts A and B of this figure show the peptide concentration obtained (with the procedure of Fig 3) with these two proteins separately, while Fig 5C shows the level of MDR1 P3 plotted against that of Na/K-ATPase P4. The regression through the points has a slope of 0.65, indicating that on a mole-for-mole basis, these cells membranes contain three molecules of the sodium pump for every two MDR1 molecules.

**Figure 6:**
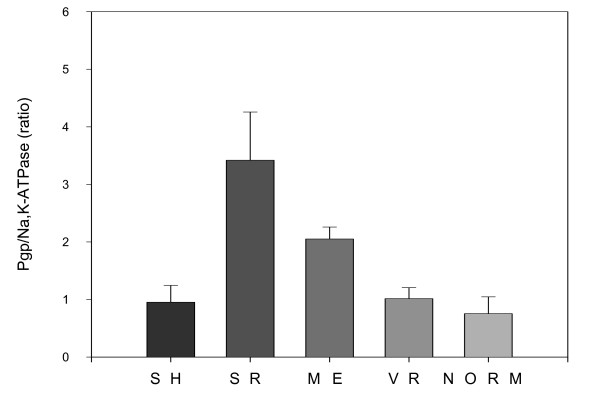
**Ratio of MDR1 P3 to Na/K ATPase P4 in white blood cell preparations from leukemia patients and normal controls**. The average of at least 5 independent experiments is shown here. The amounts of the proteins were determined with the Peptidomatrix protocol and the ratios calculated in each experiment. In order to combine the experiments the data were normalized for one of the samples (VR, taken as reference).

Fig 6 shows a summary compilation of data from this and five other experiments where this internal calibration protocol was applied to white blood cell samples taken from five different patients. In all these experiments, duplicate samples of cells from four or five patients were assayed on the same ELISA plate for both proteins. The ratio of P-glycoprotein polypeptide to the Na, K-ATPase polypeptide was computed for each patient sample and this ratio normalized to that of a particular reference sample (the cells from patient VR, mean ratio of PgP P3/ATPase P4 = 0.714 ± 0.237) for displaying in Fig 6. The amount of MDR1 is significantly higher (at p < 0.05) in the sample from patient SR than in those from patients VR and SH, and the normal donor, but not in that from ME. In one experiment of the series, a sample of the Col1000 drug-resistant cells was included on the ELISA plate. The ratio of MDR1 P3 peptide to ATPase P4 peptide in this sample was 13 times higher than that for the reference VR cells depicted in Fig 6. These cells, just like other cell lines, seem to have a very low level of Na, K-ATPase, which may not be a good normalizing control in this case.

To test the validity of the Peptidomatrix protocol we compared the results obtained with an independent method of testing for MDR levels: a clinically established functional method based on the uptake of the dye Rhodamine 123 (rho123), used on clinical samples. In cells that express functional PgP, rho123 is rapidly and actively pumped out of the cell and, within a short period of time, marked differences in intracellular fluorescence are seen between cells that have or have not extruded rho123 via this ATP-dependent pump[11]. PgP-reversing drugs, like Verapamil, can be used in the rho123 retention assay to preferentially block PgP function[12].

The clinical samples were either fresh samples or frozen samples of white blood cells. All the lymphocyte preparations were from peripheral blood or from leukopheresis treatment. The frozen samples had been kept at -70°C for up to six years and the Rhodamine uptake assay had been performed and recorded at the time of the collection.

Multidrug sensitive CEM cells (wt) and resistant CEM cells (col) served as controls. Because counting cells in the frozen samples was not possible, the parameter for the number of cells that was used was the concentration of protein. The protein concentration was measured using the Bradford method before the lysis of the cells and precipitation of the protein. The MDR protein levels in the samples were determined by the Peptidomatrix using the MDR P3 peptide and are expressed as ng peptide per mg protein.

Table 2 shows a comparison of the results obtained from the Peptidomatrix with those from the functional analysis. Care was taken to ensure that the dates of the samples that had been tested by the functional assay and the frozen samples tested by the Peptidomatrix assay were matched, because of the known variation of the expression of the protein in the same patient at different times.

**Table 2:** Summary of presence of P-glycoprotein in patient samples, as detected with the Peptidomatrix and the Rhodamine uptake assay.

	Patient	Type of leukemia	Peptidomatrix results (ng peptide/mg protein)	Peptidomatrix results (Grade)	Status by FACS (Grade)
	CEM wt		7	Negative	
	CEM col1000		114	Positive	
1	AP	AML	72.5	Positive (low)	Positive
2	DA	T-ALL	26.37	Positive (low)	Negative
3	GB	t-AML	150	Positive (high)	Positive
4	KA	T-ALL	0	Negative	Negative
5	RE	AML	144	Positive (high)	Positive
6	WE	AML	12.2	Positive (low)	Positive (low)
7	SA	AML	7	Negative	Negative
8	SN	AML	90.93	Positive (low)	Positive
9	SY	CLL	209	Positive (high)	Positive
10	WR	APL	4.9	Negative	Negative
11	YN	AML	7.9	Negative	Negative
12	CM	AML	6	Negative	Negative
13	DM	CML	62.27	Positive (low)	Positive
14	GG	AML	4.8	Negative	Negative
15	SG	AML	56.4	Positive (low)	Positive
16	OZ	AML	38.5	Positive (low)	Positive (low)

The table compares the results that we have from the clinical functional method, Rhodamine 123 uptake and the Peptidomatrix assay (see text). The Rhodamine 123 uptake assay is based on the differential dye retention in the absence and presence of Verapamil, an inhibitor of Pgp. The MDR1 protein levels in the samples were determined by the Peptidomatrix using the MDR P3 peptide and are expressed as ng peptide per mg protein. Multidrug sensitive CEM cells (wt) and resistant CEM cells (col1000) served as controls. To allow a comparison with the semiquantitative data of the other assay a gradation of MDR1 levels was defined by comparison to the control cells: The samples that had levels similar to the wt cells ± 10 % (up to 8 ng/mg protein) were graded as negative, the samples that had levels similar to the col1000 cells (100–125 ng/mg) were graded as positive, the samples that had levels higher (> 125 ng/mg) or lower then the col1000 cells (8–100 ng/mg) were graded as high-positive or low-positive respectively. The data shown are averages of duplicates, the difference between the duplicates were below 5%.

The results in Table 2 are summarized in qualitative terms (negative, low positive, positive and high positive) and their correlation was tested by using Pearson's correlation.

The Pearson's correlation between the Peptidomatrix assay and the rhodamine uptake assay was calculated as 0.79.

In order to validate the quantification of PgP by another method that is independent of the Peptidomatrix assay, PgP was also quantified by the ATPase activity assay and the results obtained by the two methods were compared.

The determination of the number of PgP molecules based on ATPase activity was performed on purified membranes obtained from CEM- col cells, using the ATPase activity associated with PgP, using both the basal activity and the verapamil (10 μM)-stimulated activity. In one typical experiment the basal ATPase activity was 23.2 mM Pi/μg protein/hour and the verapamil-stimulated activity was 82.7 mM Pi/μg protein/hour. As detailed in the Methods section the final volume of the liberated Pi determination was 260 μl. These data were converted to number of molecules/mg protein using the following turnover numbers: 2.9 s^-1 ^for the basal activity and 9.9 s^-1 ^for the verapamil-stimulated activity respectively[13]. The final calculated values were 3.48 × 10^14 ^molecules PgP/mg protein and 3.63 × 10^14 ^molecules/mg protein for the stimulated and the basal activity. The data were obtained from quadruplicate samples. To measure the PgP amount by Peptidomatrix an inhibition curve with synthetic peptide (PgP P3) was run alongside an analogous curve run with dilution of a tryptic digest of the same purified membranes used above. The Kd's (half saturation points) of the two curves were 2.87 × 10-2 μg/ml peptide and 3.11 μl digest respectively. Given that the concentration of the digest (determined prior to digestion) is 25 mg/ml, the molecular weight of the peptide is 1111 daltons, and the volume of the ELISA reaction is 100 μl, the amount of PgP in the membranes comes to 3.03 × 10^14 ^molecules PgP/mg protein, a value very close to the one obtained from the ATPase assay. In a series of experiments using different preparations of membranes, the values for the number of molecules of Pgp per mg membrane protein obtained by the two methods were 5.49 ± 1.14 × 10^14 ^(standard error n = 4) and 5.46 ± 1.73* 10^14 ^(standard error n = 4) for the ATPase (combining the data from basal and verapamil stimulated) and Peptidomatrix respectively.

**Figure 7:**
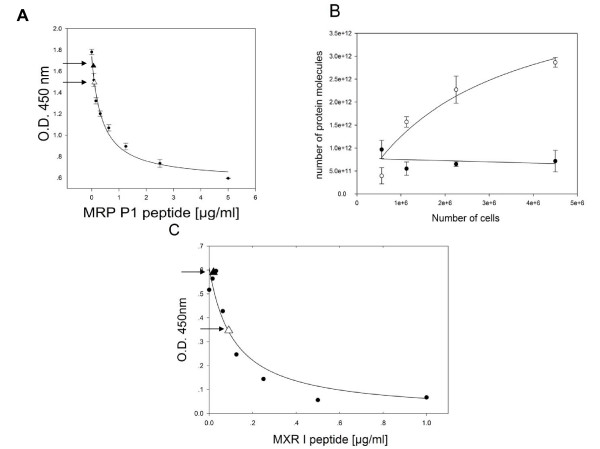
**Detection of the proteins MRP 1 and MXR**. Panel A and B show an experiment detecting the protein MRP1 in wild type and transformed MCF7 cells, using the MRP P1 peptide and one of the specific antisera. Again a calibration curve and a calculated peptide amount vs. cell amount are shown. In panel C an experiment detecting the MXR protein in wild type and transformed MCF7 cells is shown. Similarly to Figure 3A we have marked the readings of wt and resistant cell digests with a solid and an empty triangle respectively.

In fig 7 Peptidomatrix data are shown for two additional membrane proteins, MRP1, studied in a drug sensitive cell line (VP16) and the parent MCF7 cell line (Fig 7A and 7B) and MXR, studied in drug-sensitive (AdVp) and wild type MCF7 cell lines (Fig 7C). The Peptidomatrix assay shows that the parental strain is low in MRP while the drug-resistant line is rich in this protein (Fig 7B).

From the initial slope of the line drawn through the empty circles, it can be calculated that there are some 1.69 ± 0.38 million molecules of the MRP1 protein per single MCF-7 Vp 16 cell. The wild-type cell line had little if any of the MRP1 protein, by this assay.

## Discussion

The recent advances in proteomics research have fostered the emergence of many different techniques for identifying and quantitating proteins from cells and tissues. In order to apply these scientific advances to the world of routine clinical diagnostics there is a need for a simple and easy-to-use method. We present here a possible answer to this need in the form of the Peptidomatrix, and describe, as an example, its use for identifying, and assaying quantitatively, four membrane proteins present in digests of wild type and multidrug-resistant cell lines and in white blood cells of leukemia patients. The assay is ELISA-based and uses antibodies directed against small peptides that have been selected as being (i) likely to be liberated by tryptic digestion of cells and (ii) unique to the particular protein under investigation. We found that many of the peptides that we synthesized on the basis of these criteria, as being suitable for use in the Peptidomatrix protocol, did give us useful peptide-antibody combinations. Most of the peptides that were designed for use with the proteins P-glycoprotein, MRP1, and MXR gave satisfactory differences in signal when wild type and drug resistant cell strains were compared (Figs 3–4 and 6–7), suggesting that they were identifying a protein that was present in the drug resistant cells but not in the parental strains. Such a test could not be made for the peptide/antibody pairs made against the ubiquitous Na, K-ATPase. The protein for which we have most substantial data is MDR1 (also known as P-glycoprotein or ABCB1) that confers multi-drug resistance to cells in culture and is associated with drug resistance in cancer patients. For this protein, the peptide P494, the epitope of the monoclonal antibody C494, allows the specific identification of the protein in cell digests from a drug-resistant line of human T-lymphoblastic leukemia cells, the Col1000 cells. Fig 3 exemplifies the competition assay that we use in the Peptidomatrix protocol. The data from individual experiments can be transformed into plots of peptide vs. number of cells. Fig 4 summarized the results of 15 experiments using the Peptidomatrix protocol on these cells. Fitting the data by a linear hyperbola enables the initial slope of the curve to be determined. This initial slope is in units of nanograms of peptide present per million cells. Since the molecular weight of the peptide is known, the data can be translated into a number that is equivalent to the amount of P-glycoprotein molecules per cell. The number that we calculated is 10.2 million P-glycoprotein molecules per Col1000 cell. We wished to compare these values with other similar quantifications reported in the literature.

Although there have been very many studies of the expression of the MDR1 mRNA levels and of the protein in drug resistant cells, the latter using western blots or FACS analysis, we have been able to find only two quantitative studies that record the amount of P-glycoprotein present in such cells. One study[14] used the FACS technique and an antibody depletion method on three lines of mouse 3T3 cells. The highly resistant line (N3-2400) was found to have 14.2 × 10^6 ^molecules of PgP/cell[15], a value quite close to the one that we detected in the highly resistant CEM cell. The other quantitative study of P-glycoprotein molecules per cell is by Maynadie and colleagues[16]. These authors also used a FACS technique, but calibrated the FACS signal with a set of beads labeled with a fluorescent dye[17]. They measured P-glycoprotein in a sensitive and drug resistant erythroleukemic cell line (the K562 and K562-ADR cells). These cells had 291 and 303,481 P-glycoprotein molecules per cell, respectively. It must be pointed out that the K562-ADR cells are indeed resistant to doxorubicin, but much less so than the CEM cells that we used in this study and the 3T3 line mentioned previously).)[18]. Maynadie and colleagues also reported data on human leukemic cells and found very variable numbers for their P-glycoprotein content, ranging from 117 to 10,947 (mean 2509 ± 2805) molecules per cell, in the cells from the 25 donors studied[16]. Our results seem to confirm the large individual variability in the amount of PgP in white blood cells. Comparison between the Peptidomatrix method and a clinically established functional method, Rhodamine 123 uptake, shows a high correlation (Pearson's correlation of 0.79) between the two methods (Table 2). Peptidomatrix testing of MDR 1 protein in AML patients showed that 66% of them express the protein. This fits roughly with estimates in the literature, which show that 35–80 % of AML patients express the protein. This provides support for the reliability of Peptidomatrix as a diagnostic tool for identifying the presence or absence of the P-gp protein in patients.

The results obtained with Peptidomatrix were compared with several other accepted biochemical methods. Western blotting and RT-PCR, which provide only qualitative results, were fully consistent with the Peptidomatrix results (data not shown). We determined the number of P-glycoprotein molecules per cell, by measuring the ATPase activity of this protein and combining this with the known turnover number of the enzyme. This provided a quantitative result, enabling direct comparison with the Peptidomatrix. The specificity of the ATPase method for P-gp was ensured by including in the assay mixture inhibitors of all the irrelevant ATPases. The two procedures, Peptidomatrix and ATPase activity were in excellent accord. Thus, as far as can be assessed, the Peptidomatrix protocol provides a reliable method for identifying and quantitating the MDR 1 that was used in the present study.

An important point to discuss here is the general applicability of the Peptidomatrix and its ability to detect low-abundance proteins. In this study we worked with 4 different proteins, which, although similar in structure and cellular location have different sequences and distributions. The Peptidomatrix procedure, run with the appropriate peptide-antibody pair is clearly able to detect all of them. In experiments (not shown) in which we mismatched protein, peptide and antibody, we generally obtained background values and we could not build a calibration curve, indicating that the specificity of the system, at least for the range of proteins that we examined, is quite good. Virtually all the peptides chosen, listed in Table 1, were active and specific when tested, a fact that clearly indicates the broad applicability of the method. As for the sensitivity of the method we must say that there is room for improvement. The PgP experiments shown in Figure 3 and 4 indicate an LOD in the submicromolar range (10^-7^–10^-8 ^M). This is a very high value, which may not always be adequate, although in the context of the PgP field it may be. However, a look at the data in Figure 7B shows that the LOD for MRP using the MRP I peptide and cognate antibody is in the range of 10^-14 ^M, which clearly indicates that there is the potential for an adequately sensitive assay.

The proteins we studied were chosen merely on the grounds of this laboratory's familiarity with them. We offer the Peptidomatrix procedure, however, as a general solution to the problem of finding a simple and easily set up method for such assays in many biological research and clinical situations. All it requires is selecting suitable peptides in the proteins to be assayed and preparing antibodies against them. Once these are available, reliable estimates of the amounts of the specified protein can be made from digests of a variety of biological material.

## Conclusion

We have devised a simple, ELISA-based proteomics assay that enables the quantitation of designated proteins in a cell or tissue sample, and that can be used in any laboratory, with minimal specialized equipment.

## Methods

### Peptide selection

The peptides on which the ELISA is based are chosen using a three-fold screening process: First, a list is made of all the peptides that are likely to be present in a tryptic digest of the protein to be identified. This can be done using a simple word processing program or more specialized software. Next, peptides are selected that have lengths of between 7 and 15 amino acids. Each peptide from this selection is checked for its uniqueness amongst all the polypeptide chains that comprise the human proteome, using the BLAST program (parameters: PAM 30 Gap Costs Existence 5, Extension 2). The main criterion for this stage of selection is the presence of matches to other known proteins that are 5 amino acids or shorter. From this limited list, we chose the more hydrophilic peptides as being those that would be most likely to be good antigens [20,21] A number of these selected peptides were ultimately chosen for production of antibodies, and then used in the protocol described below

The peptides chosen for this investigation are shown in Table 1.

### Antibodies

The polyclonal antibodies used throughout this study were generated in rabbits by Affinity Bioreagents, Inc. (ABR, Golden, CO). The antigen for immunization was also prepared by ABR, including the synthesis of the peptides, the conjugation to a carrier and the injection to rabbits. The rabbits were bled once before immunization and 3 or 4 times after immunization. The titers were recorded and the various bleedings kept and used for the development of the immunoassay.

Monoclonal antibody C494 was purchased from Dako (Glostrup, Denmark)

### Cells

CEM (WT; Col1000.) These cells are described in Kohler and Stein [19].

CEM cell lines were grown in suspension in a 5% CO_2 _atmosphere at 37°C using RPMI-1640 medium (Biological Industries, Kibbutz Beit Ha'emek, Israel) supplemented with 10 % fetal calf serum, 2 mM glutamate, 100 U ml^-1 ^penicillin, 100 μg ml^-1 ^streptomycin, 250 μg ml^-1 ^amphotericin. The human lymphoblastoid cell line CEM Col1000 was derived from the line CEM ADR5000 by growing the original cells over four weeks in medium with 1000 ng ml^-1 ^colchicine. They were maintained in this concentration of colchicine.

MCF7 cells are human breast cancer cells. The two drug-resistant lines, VP-16, and AV, were generated by transforming them with plasmids expressing MRP1 and MXR respectively. The cells were grown in DMEM medium supplemented with fetal calf serum and antibiotics as described above. The growth medium of the VP-16 line was supplemented with 4 μM etoposide while the AV line was grown in the presence of 5 μg/ml verapamil and 3 μg/ml adriamycin.

### Peripheral blood lymphocytes

Peripheral blood mononuclear cells were separated over Ficoll gradients. Ficoll separation was performed in most cases on the same day, and when this was not possible, whole blood was kept at 4°C and separated the following day. Cells were suspended in RPM1 1640 with 10% fetal calf serum until analysis, generally on the same day or up to 48 h later.

### Sample preparation

Pellets of 20 million CEM or white blood cells (or 5 million MCF7 cells) were dissolved by re-suspending the sample in 250 μl 2% SDS in PBS and incubated at room temperature for 10 minutes. The protein was precipitated by adding 4 volumes of methanol/acetic acid pH 4.0 (15 ml methanol/40 μl glacial acetic acid) and incubating overnight at -20°C. The protein was then pelleted by centrifugation at 13,000 rpm, in an Eppendorf centrifuge (16,000 g) for 20 min at 4°C. The pellets were washed twice with ethanol and air-dried before re-suspension.

#### Protein digestion

To the protein pellet were added 70 μl of a solution containing 0.03 M Tris HCl, 2 mM CaCl_2_, and 10 μg/ml DNAase I. After incubation for 10 minutes at 37°C, 130 μl of a solution containing 2 M urea, 0.05% SDS and 0.02% NaN_3 _were added. After incubation for 10 additional minutes at 37°C, 20 μl of a stock solution of TPCK-treated Trypsin (SIGMA) (40 mg/ml) was added and the reaction mix was incubated for 20 hr at 37°C. The trypsinolysis was stopped by boiling the digest for 10 minutes. In parallel, a stock of digestion buffer, containing all the components but no protein samples, was prepared, to be used as diluent in the assay.

### ELISA

Maxisorp ELISA plates (NUNC, Denmark) were coated overnight at 4°C, with 100 μl of a0.1–2 μg/ml solution of the relevant synthetic peptide in 0.1 M carbonate buffer pH 9.6 and blocked 2 hours at room temperature with 200 μl blocking buffer (3% BSA/0.05% Tween 20 in phosphate buffered saline, PBS: 0.01 M phosphate buffer, 150 mM NaCl, pH 7.2). Serial binary dilutions of the synthetic peptide (from 5 to 0.078 μg/ml) and a blank sample were prepared in 50% digestion buffer containing 1 × PBS, 3% BSA, 0.05% Tween 20 and the desired immune serum diluted 1:3000. When monoclonal antibody C494 was used it was diluted to 0.1 μg/ml. These solutions were used for the calibration curve. The experimental samples contained 50 % cell digest, antibody as above and no additional peptide. 100 μl of these solutions were added to the wells and incubated at room temperature 1–3 hours. The wells were washed 4 times with 1 × PBS/0.05% Tween 20 and then 100 μl of horseradish peroxidase(HRP)-conjugated secondary Ab (anti-mouse or anti-rabbit) diluted 1:10,000 or 1:20,000 in blocking buffer were added in each well. After incubation for one hour at room temperature and washing, as above, the bound HRP conjugate was detected by adding 100 μl of tetramethyl benzidine. The peroxidase reaction was stopped after 5 minutes by the addition of 50 μl 0.5 M H_2_SO_4_. Optical densities at 450 nm were measured using an ELISA reader.

### Data determination

The data from the Elisa plate reader are fed into a data analysis template in the program Sigmaplot (SPSS, Chicago, IL). A plot of the OD450 vs. the concentration of the antibody or the concentration of the free peptide is drawn. The plot is then fitted, using a regression program, to a hyperbola fitting an ascending (Equation. 1) or a descending (Equation. 2) hyperbolic 3-parameter equation, as described in the text:





where Amax is the calculated maximal amplitude of the curve, D the predicted minimum of the ELISA readings, corresponding essentially to the background signal, S the concentration of the antibody or the attached peptide (in equation 1) or the free peptide (in equation 2) and Kd is the concentration that gives one-half of the shift between maximum and minimum readings.

### Rhodamine uptake

Peripheral blood mononuclear cells, prepared as described above, were stained with rhodamine-123 using the method outlined by Chaudhary and Roninson [22]. We used 150 ng/ml of rhodamine-123 for staining, for 15 min at 37°C with an efflux time of 2 h; 10 mM verapamil was used as an MDR1 inhibitor. Cells were counterstained with propidium iodide immediately prior to analysis to identify dead cells (if any were present), which were removed from the analysis by gating. We defined a positive result as one in which the Kolmogorov-Smirnov (abbreviated K-S) D value [23] was equal to or greater than 0.15 [24-26].

### Plasma membranes preparation

The cells were harvested in a table top centrifuge at 1000 rpm × 5 min, and washed twice with 20 mM Hepes pH = 7.4/0.9% NaCl buffer. The cells were then resuspended in lysis buffer (10 mM Hepes-Tris pH = 7.4, 2 mM DTT, 5 mM EDTA, 5 mM EGTA) containing protease inhibitors (cocktail from Sigma) at a concentration of 30 millions/ml. The cells were incubated in ice for 15 minutes and all subsequent procedures were performed at 4°C. The cells were homogenized in a Teflon-glass homogenizer (40 strokes), until 70% disruption. The lysis of the cells was examined under the light microscope. The homogenate is centrifuged for 10 minutes at 3000 rpm (300 g) in a tabletop centrifuge to spin down nuclei and unlysed cells. Subsequently the mitochondria were removed by centrifugation at 5500 rpm (4000 g) in a Sorvall centrifuge (rotor SS34) for an additional 10 minutes. The supernatant was then transferred to an ultracentrifuge tube and centrifuged at 35,000 rpm (114,000 g) in a 70 Ti rotor for 45 min. In this third and final centrifugation the plasma membranes were sedimented. The supernatant was discarded, the membrane pellet was resuspended in 500–1000 μl of lysis buffer and homogenized by aspiration 5 times through a 22 gauge syringe. The isolated membranes were stored at -70°C in aliquots.

### ATPase assay

The PgP-associated ATPase was determined colorimetrically as the vanadate-sensitive release of inorganic phosphate from ATP hydrolysis (REF). 1 μg membrane protein were incubated in ATPase buffer (50 mM KCl, 2.5 mM MgSO4, 0.5 mM EGTA, 3 mM DTT, 3 mM ATP, 2 mM oubain, 3 mM sodium azide, 25 mM Tris/HCl pH 7.4) and verapamil (10 μM), if desired. The reaction was carried out in 96 well microtiter plates in a final volume of 60 μl in the presence or the absence of 0.25 mM sodium orthovanadate. The microtiter plate was incubated at 37°C for one hour and the reaction was terminated by adding 200 μl of stop solution (0.2% ammonium molybdate, 1.3% sulfuric acid, 0.9% SDS and 1% ascorbic acid). After an additional 30 minutes of incubation the absorbance at 620 nm was read in a microplate reader. ATPase activity was expressed in mmol Pi/hr/mg protein.

## Competing interests

The author(s) declare that they have no competing interests.

## Authors' contributions

HG and J B-S initially developed the Peptidomatrix procedure. J B-S worked out the method for preparations of tryptic peptides from biological material. J B-S, OB, M K-G and HG performed the experimental work and contributed to the discussions. OB, WDS and UP wrote the manuscript. DGR and UP provided intellectual guidance. DGR provided the patient material. WDS initiated the project, developed the mathematical analyses used, and directed the research.
